# Comment on “A re-assessment of the safety of silver in household water treatment: rapid systematic review of mammalian in vivo genotoxicity studies”

**DOI:** 10.1186/s12940-017-0334-1

**Published:** 2017-11-13

**Authors:** Daniele Lantagne, Justine Rayner, Anjuliee Mittelman, Kurt Pennell

**Affiliations:** Tufts University, Civil and Environmental Engineering, 200 College Avenue, Medford, MA 02155 USA

**Keywords:** Ceramic filters, Elution, Genotoxicity, Silver ions, Silver nanoparticles, Water treatment

## Abstract

We wish to thank Fewtrell, Majuru, and Hunter for their article highlighting genotoxic risks associated with the use of particulate silver for primary drinking water treatment. The recent promotion of colloidal silver products for household water treatment in developing countries is problematic due to previously identified concerns regarding manufacturing quality and questionable advertising practices, as well as the low efficiency of silver nanoparticles to treat bacteria, viruses, and protozoa in source waters. However, in the conclusion statement of the manuscript, Fewtrell *et al.* state, “Before colloidal Ag or AgNP are used in filter matrices for drinking water treatment, consideration needs to be given to how much silver is likely to be released from the matrix during the life of the filter.” Unfortunately, it appears Fewtrell *et al.* were unaware that studies of silver nanoparticle and silver ion elution from ceramic filters manufactured and used in developing countries have already been completed. These existing studies have found that: 1) silver ions, not silver nanoparticles, are eluted from ceramic filters treated with silver nanoparticles or silver nitrate; and, 2) silver ions have not been shown to be genotoxic. Thus, the existing recommendation of applying silver nanoparticles to ceramic filters to prevent biofilm formation within the filter and improve microbiological efficacy should still be adhered to, as there is no identified risk to people who drink water from ceramic filters treated with silver nanoparticles or silver nitrate. We note that efforts should continue to minimize exposure to silver nanoparticles (and silica) to employees in ceramic filter factories in collaboration with the organizations that provide technical assistance to ceramic filter factories.

## Background

We wish to thank Fewtrell, Majuru, and Hunter [1] for their article highlighting genotoxic risks associated with the use of particulate silver for primary drinking water treatment. The recent promotion of colloidal silver products for household water treatment in developing countries is problematic due to previously identified concerns regarding manufacturing quality and questionable advertising practices [2], as well as the low efficiency of silver nanoparticles to treat bacteria, viruses, and protozoa in source waters [3]. Potential genotoxic impacts of silver nanoparticles represent an additional concern, and highlights the need to carefully consider their use for household drinking water treatment in developing countries.

However, in the conclusion statement of the manuscript, Fewtrell *et al.* [1] state, “Before colloidal Ag or AgNP are used in filter matrices for drinking water treatment, consideration needs to be given to how much silver is likely to be released from the matrix during the life of the filter (e.g., work by Garboś and colleagues).” Unfortunately, the work completed by Garboś was not conducted in matrices similar to the ceramic filters manufactured in developing countries and the authors appear to be unaware that studies of silver nanoparticle and silver ion elution from ceramic filters manufactured and used in developing countries have already been completed [4].

## Main text

Ceramic water filters are locally manufactured in more than 50 countries worldwide [5], are considered one of the most promising household water treatment methods [6], and application of silver nanoparticles (nAg or AgNP) or silver nitrate (AgNO_3_) has been shown to reduce biofilm formation within, and improve the microbiological efficacy of, ceramic water filters [7]. There are benefits and drawbacks to either form of silver applied to ceramic filters; silver nitrate can be purchase locally and is less expensive, while silver nanoparticles must be imported. However silver nitrate elutes more quickly, and silver concentrations in treated drinking water are more likely to exceed World Health Organization guidelines if applied at concentrations sufficient to improve disinfection performance [4, 7]. Thus, silver nanoparticles are recommended for use in ceramic filter factories [5].

In the Mittelman *et al.* ^*3*^ study, the impacts of nanoparticle detachment, dissolution, and cation exchange on silver elution was investigated as a function of influent water pH (5–9), ionic strength (1–50 mM), and cation species (Na^+^, Ca^2+^, Mg^2^) from filter disks painted with 0.03 mg/g casein-coated (nAg) or (AgNO_3_). Under all conditions and regardless of the applied silver form, silver elution was controlled by the release of Ag^+^ and subsequent cation exchange reactions within the ceramic filter. Overall, > 99% of silver was eluted as dissolved Ag^+^ form rather than in the nanoparticle (nAg) form.

Thus, silver nanoparticles were not directly released into drinking water from ceramic filters impregnated with nAg, rather Ag^+^ eluted from ceramic filters (regardless of whether they were painted with silver nanoparticles or silver nitrate solution). Since all 16 studies identified in the Fewtrell et al. [1] review focused on the genotoxic risk of silver nanoparticles (nAg), rather than the risk from silver ions, the conclusions of the Fewtrell et al. [1] are not applicable to exposure from drinking water treated with locally-manufactured ceramic filters impregnated with silver nanoparticles or silver nitrate.

Additionally, a recent study by Li *et al.* [8] examined the mechanisms of genotoxicity of nAg and Ag^+^, using a mammalian cell micronucleus assay. Their work incorporated gene expression analysis, measurements of oxidative stress, and the use of a reactive oxygen species scavenger and a chelator to evaluate the role of Ag^+^ in the genotoxicity of nAg. The authors found that silver ions (Ag+) did *not* release hydroxyl radicals and concluded: “These results suggest that, although both AgNPs and Ag^+^ can cause genotoxicity via producing oxidative stress, the mechanisms are different and the nanoparticles, but not the released ions, are mainly responsible for the genotoxicity of AgNPs.”

Thus, the primary exposure to silver nanoparticles with regards to ceramic filters is to employees during production, not users drinking the water. In ceramic filter factories, silver is imported in either a powder or concentrated liquid form [5]. Employees typically prepare a concentrated silver solution once per week or month, depending on production, and dilute the solution as needed for application to ceramic filters. It is currently recommended that employees wear N95 masks to reduce exposure when preparing powdered or liquid silver nanoparticles, and wear gloves when applying dilute solution onto filters [5]. Fewtrell *et al.* [1] are correct in stating that “health and safety precautions are not strictly adhered to in the production of ceramic filters in low income countries” [9] and while the only study included in the Fewtrell *et al.* [1] paper that evaluated exposure via inhalation found no difference, maintaining precautionary administrative controls and personal protection equipment use to minimize exposure when handling silver is still advised. Additionally, the key health risk of silica exposure during clay processing for employees in ceramic filter factories should also be ameliorated with personal protective equipment [10].

## Conclusions

In summary, based on existing data that Fewtrell *et al.* [1] did not reference: 1) silver ions, not silver nanoparticles, are eluted from ceramic filters treated with silver nanoparticles or silver nitrate; 2) silver ions have not been shown to be genotoxic; 3) the existing recommendation of applying silver nanoparticles to ceramic filters to prevent biofilm formation within the filter and improve microbiological efficacy should still be adhered to; and, 4) efforts should continue to minimize exposure to silver nanoparticles (and silica) to employees in ceramic filter factories in collaboration with the organizations that provide technical assistance to ceramic filter factories.

## Abbreviations

Ag +: Silver ions
AgNO_3_: Silver nitrate
AgNPor nAg: Silver nanoparticles
