# Supplementary material for: Comment on “A re-assessment of the safety of silver in household water treatment: rapid systematic review of mammalian in vivo genotoxicity studies”
Source: Environ Health. 2017 Nov 13;16:121. doi: 10.1186/s12940-017-0334-1 (PMC5684740; doi:10.1186/s12940-017-0334-1)
Supplement: Related texts

# Reply: Author’s response to: Comment on “A re-assessment of the safety of silver in household water treatment: rapid systematic review of mammalian in vivo genotoxicity studies”

**Authors:** Paul R. Hunter, Batsirai Majuru, Lorna Fewtrell

**Affiliations:** 2 0000 0001 1092 7967 grid.8273.e Norwich Medical School, University of East Anglia, Norwich, NR4 7TJ UK; 3 0000000121682483 grid.8186.7 Centre for Research into Environment and Health, University of Aberystwyth, Aberystwyth, UK

## Abstract

We thank Lantagne and colleagues for their comments on our recent paper. As we stated in our original paper we could find no evidence that ionic silver was associated with genotoxicity and the balance of evidence indicates that it is not. About whether ceramic filters release nanoparticles in routine use, we do not consider that the single laboratory study is necessarily sufficient to assess the risk over the lifetime of a single ceramic filter during routine use in the field. We do agree with Lantagne and colleagues that risk from occupational exposure to silver nanoparticles during production of filters in low income countries with inadequate personal protection is a significant concern.

## Keywords

SilverNanoparticlesGenotoxicityDNA damageNanocomposites

## Main text

We thank Lantagne and colleagues for their comments on our recent paper [1]. We would like to respond to two points. Firstly about the potential genotoxicity of ionic silver. We agree that ionic silver does not appear to be genotoxic and we stated this very clearly in our paper: “We did not find any papers which suggested that *in vivo* exposure to ionic silver posed a threat of genotoxicity, but those few studies that reported on ionic silver found no effect even from *in vitro* studies”. Given that the recent paper that Lantagne cites [8] was not an in vivo study either but an in vitro study, it would have not been included in our review and does not alter our conclusion in this regard.

On the issue of release of nanoparticles from silver nanocomposites. We did state that “Before colloidal Silver or Silver Nanoparticles are used in filter matrices for drinking water treatment, consideration needs to be given to how much silver is likely to be released from the matrix during the life of the filter”. Release of nanoparticles from nanocomposites is certainly a cause for concern [11, 12]. However, there are many different types of filter and we did not feel there was sufficient evidence to debate whether certain filters were more or less likely to release nanoparticles over their lifetime than others. Lantagne *et al.* cite their paper to show that in the laboratory at least it is ionic silver and not nanoparticles that is released [4]. We would, however, question whether the results of this carefully controlled laboratory based study can be extrapolated to the lifetime of a ceramic filter’s use when such filters may be subject to a range of abuse not modelled in these laboratory experiments.

Finally, we would join with Lantagne and colleagues in stressing the potential occupational risks associated with the use of silver nanoparticles in manufacturing in low income countries. The use of appropriate personal protective equipment is essential and consideration should probably also be given to the need for health screening of workers.
